# Mitogenomics of southern hemisphere blue mussels (Bivalvia: Pteriomorphia): Insights into the evolutionary characteristics of the *Mytilus edulis* complex

**DOI:** 10.1038/srep26853

**Published:** 2016-05-31

**Authors:** Juan Diego Gaitán-Espitia, Julian F. Quintero-Galvis, Andres Mesas, Guillermo D’Elía

**Affiliations:** 1 Instituto de Ciencias Ambientales y Evolutivas, Universidad Austral de Chile, Casilla 567 Valdivia, Chile; 2 CSIRO Oceans and Atmosphere, GPO Box 1538, Hobart 7001, TAS, Australia; 3 Programa de Doctorado en Ciencias mención Ecología y Evolución, Facultad de Ciencias, Universidad Austral de Chile, Valdivia, Chile

## Abstract

Marine blue mussels (Mytilus spp.) are widespread species that exhibit an antitropical distribution with five species occurring in the Northern Hemisphere (*M. trossulus*, *M. edulis*, *M. galloprovincialis*, *M. californianus* and *M. coruscus*) and three in the Southern Hemisphere (*M. galloprovincialis*, *M. chilensis* and *M. platensis*). Species limits in this group remain controversial, in particular for those forms that live in South America. Here we investigated structural characteristics of marine mussels mitogenomes, based on published F mtDNA sequences of Northern Hemisphere species and two newly sequenced South American genomes, one from the Atlantic *M. platensis* and another from the Pacific *M. chilensis*. These mitogenomes exhibited similar architecture to those of other genomes of *Mytilus*, including the presence of the *Atp8* gene, which is missing in most of the other bivalves. Our evolutionary analysis of mitochondrial genes indicates that purifying selection is the predominant force shaping the evolution of the coding genes. Results of our phylogenetic analyses supported the monophyly of Pteriomorphia and fully resolved the phylogenetic relationships among its five orders. Finally, the low genetic divergence of specimens assigned to *M. chilensis* and *M. platensis* suggests that these South American marine mussels represent conspecific variants rather than distinct species.

Molluscs of the subclass Pteriomorphia are an old and successful lineage of saltwater bivalves that includes the orders Arcoida, Limoida, Ostreoida, Pectinoida, Pterioida and Mytiloida^1^. In the latter order, the family Mytilidae, generally known as marine mussels, is an important component of rocky intertidal communities in temperate waters around the world^2^. These bivalves are among the most studied marine organisms due to their ecological and economic importance^3^, and an equally relevant role as sentinel species for pollution in coastal areas^4^. Within Mytilidae, mussels of the genus *Mytilus* are widespread species at middle and higher latitudes (Fig. 1). This group exhibits a typical antitropical distribution with five species occurring in the Northern Hemisphere (*M. trossulus*, *M. edulis*, *M. galloprovincialis*, *M. californianus* and *M. coruscus*) and three in the Southern Hemisphere (*M. chilensis, M. galloprovincialis* and *M. platensis*) (Fig. 1)^5^,^6^,^7^,^8^,^9^,^10^,^11^.

In the last decades there has been a significant increase in the taxonomic understanding of *Mytilus*, mostly prompted by the analysis of molecular evidence e.g.^12^,^13^,^14^,^15^. However, disagreements remain regarding the number and identity of the species that live in South America^16^. In addition to the now settled dispute over the presence of *M. galloprovincialis* in the coast of Chile^17^, different views remain on the distinction, at the species level, of Atlantic and Pacific populations and of these with those of the Northern Hemisphere. Some authors have suggested that mussels in the Pacific coast of South America could correspond to a Southern Hemisphere lineage of *M. galloprovincialis*^13^. However, the most accepted view relates South American forms with *M. edulis*, either as a single species or as one or two closely related species. For instance, McDonald *et al.*^18^ and Seed^19^ considered the common mussels from temperate waters of the Northern and Southern Hemispheres to be a single cosmopolitan species, *Mytilus edulis* Linnaeus, 1758. On the other hand, some authors considered South America mussels distinct at the subspecies or species level and limit *M. edulis s.s.* to the Northern Hemisphere. As such, marine mussels in Argentina and Uruguay have been referred to as *Mytilus platensis*^20^ or *Mytilus edulis platensis* e.g.^6^, whereas those found in Chile from the Tirúa River (38°S) to the Magellan Strait (53°S) as *Mytilus chilensis* (Hupé, 1854)^16^, and *Mytilus edulis chilensis*^7^. Finally, a third classificatory scheme considers Atlantic and Pacific South American populations to be distinct from those of the northern *M. edulis s.s.*, but regard them as belonging to a single subspecies, *Mytilus edulis platensis*^10^. Previous phylogenetic studies about the evolutionary relationships among *Mytilus* spp. have suggested that Southern Hemisphere mussels could be closely related to those mussels in the *M. edulis* complex of the Northern Hemisphere in which *M. edulis* and *M. galloprovincialis* are sister taxa, whereas *M. trossulus* is more distantly related^5^,^18^,^21^,^22^,^23^.

Species of the genus *Mytilus* exhibit unusual and interesting features related to the heteroplasmy of their mitochondrial DNA (mtDNA)^24^. These organisms commonly have two types of mitochondrial genomes (mitogenomes), known as M and F, in stable co-existence. The sperm contains exclusively the M genome and is paternally inherited, whilst the egg has the F type and is inherited by both male and female somatic cells and female gonadal cells^25^,^26^,^27^. Comparisons of F and M mtDNA sequences of *Mytilus* species have shown that the M mitogenome evolves more quickly than the F mitogenome^24^,^28^,^29^. From these studies, it appears that both genomes experience purifying selection, but this selection is relatively relaxed for the M mtDNA in Mytilus spp.^24^,^28^,^29^,^30^. The mitogenome represents an important potential target of natural selection in taxa that are distributed across environmental gradients^31^, such as the case of marine mussels^5^. These organisms inhabit broader latitudinal gradients of coastal marine environments (Fig. 1) with dynamic changes (e.g. fluctuations in temperature, dissolved oxygen, salinity, desiccation, UV-radiation, and chemical contaminant exposure etc.), which may impose oxidative stress to them^32^, affecting the mitochondrial respiration and leading to irreversible damage of mtDNA^33^. Non-synonymous single nucleotide polymorphisms in any of the mtDNA genes encoding enzymes of the electron transport chain (ETC) can potentially affect the quality of electron flow or influence other relevant binding sites, such as that of coenzyme Q or CoQ^34^.

In this work we investigated structural and evolutionary characteristics of marine mussels mitogenomes, based on published complete F mtDNA sequences of Northern Hemisphere species and our two newly sequenced South American marine mussels. We detail the main features of their genomic architecture and compare these features to those from other bivalves. Additionally, we investigated the phylogenetic relationships within the *M. edulis* complex and among mollusks of the subclass Pteriomorphia. Finally, we analyze the evolutionary patterns of mtDNA protein-coding genes of marine mussels in order to evaluate the nature of the selective forces acting on their mitogenomes.

## Results and Discussion

### Mitogenome architecture of *M. chilensis* and *M. platensis*

The F mitogenomes (from somatic cells) of the Southern Hemisphere marine mussels, *Mytilus chilensis* and *M. platensis*, are circular DNA molecules with a length of 16,765 bp (Table 2, Fig. 2A), which is in the range of the mitogenome size of their closely related mytilid species, and similar to those of other described marine mussels of the subclass Pteriomorphia (Supplementary Table S1). The A+T content of the two newly sequenced species represents 61.8% of the total genome whereas the G+C content represents 38.2% (Table 2, Fig. 2). These values are consistent with those described for other bivalves, with a particular disparity in A+T and G+C along the genome^35^ (Supplementary Table S1). In general, mtDNA sequences obtained from somatic tissue of *M. chilensis* and *M. platensis* exhibited uniform coverage across the entire length, lack of heteroplasmy (i.e., recombination or traces of the M mtDNA), and the absence of nuclear copies of mtDNA. These mitogenomes revealed a highly conserved genomic architecture with all 37 genes normally found in metazoans (13 protein-coding genes, 2 ribosomal RNA genes and 22 transfer RNAs)^36^, including the *Atp8* gene, which is reported as missing in other bivalve mollusks^21^,^35^. The absence of this ATP subunit is controversial as it has been detected in some freshwater mussels (Palaeoheterodonta)^37^ and saltwater clams (Heterodonta)^38^. The uncertainty related to the annotation of this protein-coding gene, could be due to the structural characteristics (e.g., short length) and extreme variability of the *Atp8* gene among bivalves^23^,^38^,^39^. Notwithstanding, according to the available data this gene has a complex pattern of presence/absence in groups such as Pteriomorphia in which some pectinoids and ostreoids possess the *Atp8* gene whereas others do not^38^. This pattern is consistent with our mitogenomic comparison among marine mussels (Mytilidae), where this gene is functional (including the presence of start and stop codons) only in species of the genus *Mytilus* and reduced, non-functional or absent in the other mytilids (Fig. 2B, Supplementary Table S1 and Fig. S1).

In addition to the protein-coding genes (PCGs), most metazoan have a set of 22 tRNA genes, including two copies of *tRNA-Leu* and two of *tRNA-Ser*^36^. However, in most bivalve mitogenomes the tRNA set involves a total of 23 genes due to the presence of two *tRNA-Met* e.g.^21^,^35^,^37^. This is in accordance with our findings in which the two *tRNA-Met* are recognized by the anticodons CAU and UAU. Each of the 23 tRNA genes folded into a typical cloverleaf secondary structure as predicted by ARWEN and tRNAscan-SE. For *M. chilensis* and *M. platensis* these genes were within the size range of 63 bp (*tRNA-Ser* and *tRNA-Thr*) to 69 (*tRNA-Lys* and *tRNA-Met*) and spread on the same strand over the entire genome (Table 2 and Fig. 2A). Similarly, the rRNA subunits (*s-rRNA* and *l-rRNA* genes) of our South American mussels are encoded on the major strand (Table 2 and Fig. 2A) and resembled those of other mytilids (Fig. 2B). Like with the tRNAs and rRNAs, all of the PCGs in our sequenced species were encoded by the major strand (Table 2 and Fig. 2A), maintaining the same transcriptional orientation of other *Mytilus* (Supplementary Fig. S2) and marine bivalve genomes^40^,^41^,^42^. Finally, within the genus *Mytilus*, three unique clusters of tRNA genes were identified between the *Cox2*-*Nadh1*, *Nadh2*-*Nadh3* and the small and large *rRNA* genes (Table 2 and Fig. 2A). These clusters are preserved due to the highly conserved gene order of this genus. Among the five orders of Pteriomorphia species that are currently reported at the NCBI (Table 1), only few gene blocks are shared between any two pairs (Supplementary Fig. S2); most of the rearrangements are switches of tRNA genes, which change their position more frequently than PCGs and rRNA genes^43^,^44^.

Mitochondrial genomes are characterized by the presence of a large AT-rich intergenic region that contains the putative origin for mitochondrial DNA replication (POR)^43^,^45^. In our sequenced species, this region is located between the *l-rRNA* and *tRNA-Tyr* genes, with a length of 1,173 pb (Table 2 and Fig. 2A), which is in the range size of those observed among closely related species^22^,^23^,^26^,^46^, and almost twice longer than that of other Pteriomorphia species^35^,^42^. This difference in the POR size could be explained by deletions or duplications of tandem sections^35^,^43^ or by the high rate of divergence of the variable domains of this region reported for the genus Mytilus^47^. As can be seen in the CCT BLAST map, the low degree of conservation of this mtDNA region is a consequence of the dynamic nature of the control region (Fig. 2B).

### Evolutionary patterns of protein-coding genes (PCGs)

To evaluate the nature of the selective forces acting on marine mussels mitogenomes, we estimated the synonymous (dS) and non-synonymous substitution (dN) rates of PCGs. The dN/dS ratio is a simple measure of selective pressures acting on gene that indicates neutral mutation (dN/dS = 1), negative or purifying selection (dN/dS < 1) and positive or diversifying selection (dN/dS > 1)^48^. Our dN/dS evolutionary analysis of the concatenated alignment of PCGs, revealed that out of the 3973 codons, 3360 are subject to negative selection and 14 showed signatures of positive selection by at least one of the implemented methods (Fig. 3A; Supplementary Table S2). Codons under positive selection belong to the *Cytb*, *Nadh4*, *Atp8*, *Nadh5* and *Nadh6* genes (Fig. 3A; Supplementary Table S2). Similar results have been reported in other marine mussels in which an accelerated accumulation non-synonymous substitutions was detected only at terminal branches of the phylogenetic trees^22^. This finding was also consistent with our GA-Branch analysis, particularly for the clade that includes all of the representative species of the *M. edulis* complex (Figure not shown). The overall picture of evolutionary rates of mitogenomes in marine mussels indicates that purifying selection is the predominant force shaping the evolution of marine mussels PCGs (Fig. 3B), with dN/dS ratios between 0.096–0.262 (Supplementary Table S3). The lowest dN/dS was detected for the *Cox1* gene (Fig. 3B), which is in agreement with previous findings in *M. edulis* and *M. galloprovincialis*^22^,^23^,^26^ and may be explained by the highly conservative nature of this gene, as observed in other mollusks e.g.^43^. It has been suggested that genes with the lowest dN/dS values are likely to be evolving under the strongest selective constraint, whereas those with the highest may be evolving in response to positive selection or relaxed constraint^48^.

### Phylogenetic analyses

**Figure 1:**
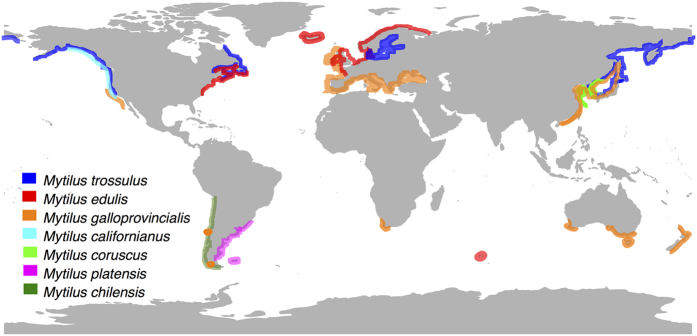
Geographic distribution of marine mussels of the genus Mytilus. Approximate distributions of mussels were compiled from various sources, including^5^,^6^,^7^,^8^,^9^,^10^,^11^. The map was generated with the R package “maps” and modified using Inskcape v.0.91 (http://inkscape.org/).

**Figure 2:**
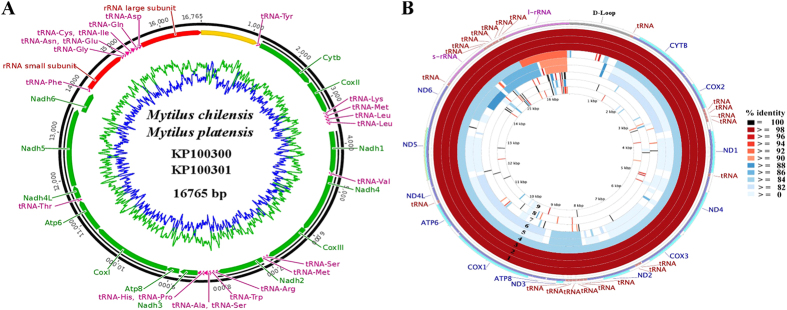
(**A**) Graphic representation of the mitocondrial genome architecture of *Mytilus chilensis* and *Mytilus platensis*. AT and CG content and their changes throughout the genome are represented in blue and green, respectively. The control region is located in the yellow segment. (**B**) Graphical map of the BLAST results showing nucleotide identity between *M. chilensis* and the other 9 species of the order Mytiloida listed in Table 1, as generated by the CGView comparison tool (CCT). CCT arranges BLAST result in an order where sequence that is most similar to the reference (*M. chilensis*) is placed closer to the outer edge of the map. The rings labelled 1 to 9 indicate BLAST results of *M. chilensis* mitogenome against *M. platensis*, *M. edulis*, *M. galloprovincialis*, *M. trossulus*, *M. coruscus, M. californianus, P. viridis, B. exustus, M. senhousia*, respectively.

**Figure 3:**
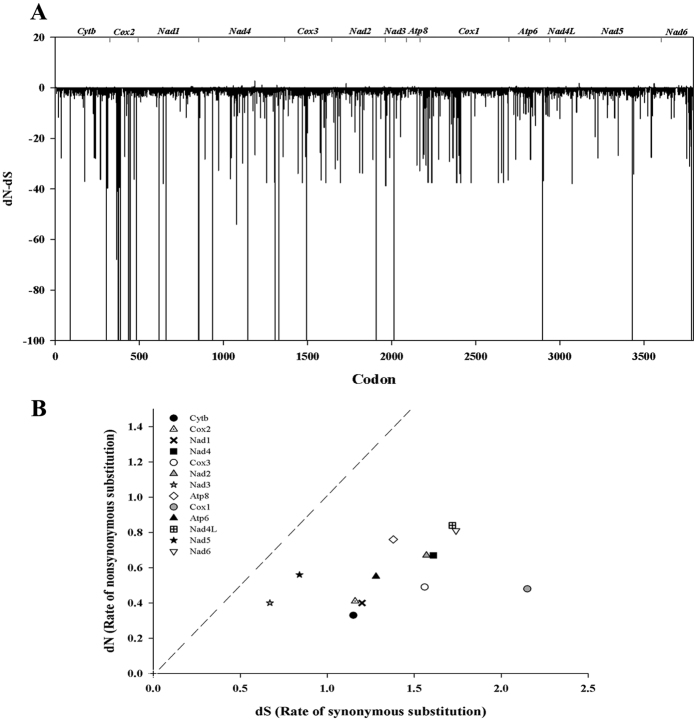
(**A**) Codon sites of PCGs under positive or negative selection. The vertical axis represents normalized dN/dS capped at −100 and the horizontal axis represents codon position. The values above zero indicate a candidate for positively selected sites. The gene position is shown at the top. (**B**) Evolutionary rates of mitochondrial protein coding genes in marine mussels. Rate of n ucleotide substitutions causing amino acid changes (dN) is plotted relative to substitutions at silent sites (dS) for each region. The dotted line indicates the theoretical expectation of neutral evolution (dN = dS). The area below the dotted line represents purifying selection (dN < dS).

**Figure 4:**
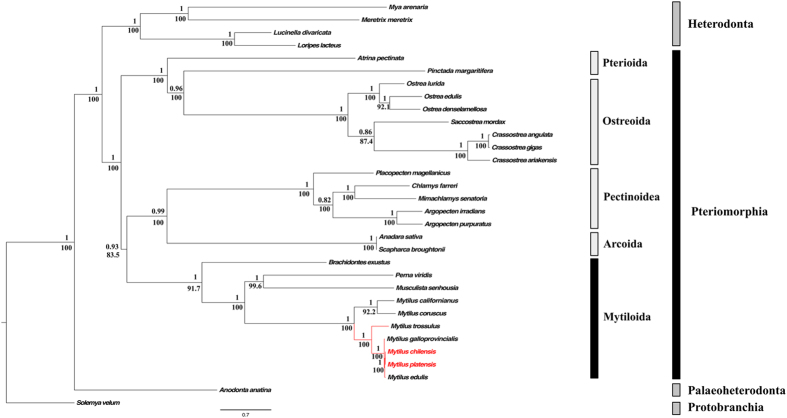
Maximum likelihood tree of the nucleotide sequences of 12 protein coding genes of marine mussels (Subclass Pteriomorphia). The numbers of the nodes show the Bayesian posterior probabilities and maximum likelihood bootstrap percentages.

Based on the concatenated alignment of 12 PCGs of the bivalve species used in this study (Table 1), our Best Partition Scheme (BPS) analysis generated seven subset partitions (Supplementary Table S4). This BPS and selected models of molecular evolution were used for both Bayesian (BI) and Maximum Likelihood (ML) analyses, which produced identical topologies with similar branch lengths and strong bootstraps (ML analysis) and posterior probabilities (Bayesian inference) values (Fig. 4). As expected, all of the representative species of the family Mytilidae form a clade (Fig. 4). Within this group, *M. chilensis* and *M. platensis* were recovered in the same clade (Fig. 4) that coupled with the high similarity (Pairwise distance = 0.002 ± 1 × 10^−6^; Mean ± SD, Supplementary Table S5) of their mitogenomes (Fig. 2A) suggest that *M. chilensis* and *M. platensis* represent conspecific variants rather than distinct species. These results were also supported by our species delimitation analysis, in which the values of the ratio between the average distance of *M. chilensis-M. platensis* (Intra_Dist) and the average distance between them and their closest species of *Mytilus* (Inter_Dist) were below 0.15. The low Intra_Dist/Inter_Dist is indicative of small genetic differences between the newly sequenced mitogenomes relative to the differences observed when those are compared with other species of *Mytilus*, meaning that *M. chilensis-M. platensis* belong to a single species^49^. Previous ecological and physiological studies have suggested a sympatric distribution for *M. chilensis* and *M. platensis* in the southern limit of their latitudinal distribution (Fig. 1)^6^. However, our results and those of other studies using allozymes^10^,^50^,^51^, have suggested that the same lineage of species level inhabit the Pacific and Atlantic coasts of South America. For the moment we regard this lineage as a distinct species of *M. edulis s.s.* and as such refer to it as *M. platensis*. Future taxonomic assessments would test our taxonomic hypothesis. In this regard, future analysis should include the analyses of nuclear DNA sequences to discard an eventual case of mitochondrial introgression causing the similarity of the mitogenomes of *M. chilensis* and *M. platensis*.

According with our results, *M. platensis* shares a most recent common ancestor with *M. edulis sensu stricto*, followed by *M. galloprovincialis* and *M. trossulus*, supporting the monophyly of the *M. edulis* complex (Fig. 4). The clade of the blue mussels was recovered as sister to the clade containing the northern Pacific California (*M. californianus*) and the Korean (*M. coruscus*) mussels (Fig. 4), which is congruent with previous phylogenetic works with complete mitochondrial genomes^22^,^23^.

Our phylogenetic analyses clearly supported the monophyly of four orders of Pteriomorphia bivalves (Mytiloida, Arcoida, Pectinoidea, and Ostreoida) (Fig. 4) as reported in other molecular studies using nuclear markers and morphological data^1^,^52^,^53^,^54^. However, limited taxonomic sampling precludes testing the monophyly of Pterioida, the fifth order of pteriomorphs (Table 1). Overall, the ML and BI analyses recovered a strongly supported sister relationship between a clade containing Pectinoidea + Arcoida and the Mytiloida lineage (Fig. 4). In addition, Ostreoida appeared as sister to Pterioida (Fig. 4) in agreement with previous analysis based on the *18S* rDNA and partial sequences of the *Cox1*^1^,^54^. Nevertheless, our results did not support the “hard politomy” reported by several authors within the subclass Pteriomorphia that could reflect a true rapid radiation dated about 490 Mya^1^,^52^,^54^. This discrepancy could be explained by the different methodologies implemented and in particular for the distinct character sampling. In this study, in addition to assess the variation at a mitogenomic scale, we used a methodology that concatenates PCG sequences and sets the best model of evolution for each codon position within each of the PCG genes; this combination allowed us to fully resolve the phylogenetic relationships among orders of Pteriomorphia.

Finally, in our phylogenetic reconstruction, the overall picture of the relationships among major bivalve lineages is in agreement with the multigene phylogenomic view of an early branching of the lineage leading to Protobranchia^55^ followed by that of Palaeoheterodonta (Fig. 4)^38^, with Heterodonta and Pteriomorphia in a sister group relationship^38^,^55^. Nonetheless, in a recent and more robust molecular study, Palaeoheterodonta was recovered as sister taxa to Heterodonta, both forming a clade sister to Pteriomorphia^56^. The topological incongruence could be due to the low number of species of Palaeoheterodonta used in our phylogenetic analyses.

## Conclusion

In the present study, we determined the complete mitochondrial genome sequences of the marine mussels *Mytilus chilensis* and *M. platensis*, the first completely sequenced and annotated genomes of the Southern Hemisphere representative species of the family Mytilidae. These mitogenomes exhibited similar characteristics in their architecture and gene order to those of other genomes of *Mytilus*, including the presence of the *Atp8* gene, which has been mentioned as missing in other bivalves. However, several gene rearrangements were detected within Mytilidae and among species of the five orders of Pteriomorphia. Our analysis indicates that purifying selection is the predominant force shaping the evolution of the marine mussels mitochondrial PCGs. This observation is consistent with the central role that the typical 13 mtDNA-encoded protein products play in fundamental biological process of cellular respiration. Results of our phylogenetic analyses supported the monophyly of Pteriomorphia and placed the order Mytiloida in a sister group relationship with a clade containing the orders Pectinoidea and Arcoida. Finally, the low genetic divergence of *M. chilensis* and *M. platensis* coupled with the high similarity of their mitogenomes suggest that these South American marine mussels represent conspecific variants rather than distinct species, to which the name *M. platensis* applies. The mitogenomes reported here can provide basic information to studies investigating aspects of phylogeography, systematics and climate change ecophysiology of these economically important marine species.

## Methods

### Ethics statement

This study was carried out in strict accordance with the recommendations in the Guide for the Care and Use of Laboratory Animals of the Comisión Nacional de Investigación Científica y Tecnológica de Chile (CONICYT). All experiments were conducted according to current Chilean law. The protocol was approved by the Committee on the Ethics of Animal Experiments of the Universidad Austral de Chile.

### Species collection and DNA isolation

Adult mussels of *Mytilus chilensis* and *M. platensis* were collected from the intertidal and subtidal zones in the Pacific coast of South America (Valdivia: 39°46′S–73°29′W; and Chiloe: 43°08–73°12′W) and South Atlantic waters (Mar del Plata: 38°10′S–57°27′W), respectively. Sex was determined by gonad examination under a microscope for the detection of sperm and eggs. DNA was extracted by the isolation of intact mitochondria from approximately 150 mg of fresh tissue (somatic cells from mantle skirt and the adductor muscle) from ten female mussels of each locality using the Mitochondrial Isolation Kit (Thermo Scientific). The isolated mitochondrial pellet of each mussel was used for the mtDNA extraction by mean of the Mitochondrial DNA Isolation kit (BioVision).

### Mitochondrial genomes sequencing, assembly and annotation

Shotgun libraries of *M. chilensis* and *M. platensis* were sequenced using a combination of 454 (Roche Genome Sequencer GS FLX Titanium) and Sanger sequencing technologies on ABI 3730XL sequencers by Eurofins MWG Operon (Huntsville, USA). DNA samples were nebulized, individually bar-coded to perform emulsion-based clonal amplification (emPCR) and sequenced to approximately 20-fold coverage. Raw reads of *M. chilensis* (6315, average length = 413.3 bp, Q-score = 29.3, Coverage ≥10 = 99.6%) and *M. platensis* (5832, average length = 401.7 bp, Q-score = 29.8, Coverage ≥10 = 98.7%), were proof read, separated, and assembled, according to the bar-codes, into contigs in Celera Assembler v.6.1. Assembly data was evaluated with the statistical overview and quality scoring files of each single read. For both species, reads were assembled in a single contig with uniform coverage distribution. All nucleotide sequences obtained in this work were deposited in the NCBI Genbank repository. Accession numbers can be found in Table 1.

Mitochondrial DNA sequences (i.e., protein-coding genes, rRNAs, tRNAs and noncoding regions) were identified by BLAST searches at NCBI with BLASTn and BLASTx^57^ using the default values of all algorithm parameters (http://www.ncbi.nlm.nih.gov). In addition, protein-coding genes (PCGs) were identified with the ORF Finder at NCBI using the invertebrate mitochondrial genetic code. The limits of both protein-coding and ribosomal RNA genes were adjusted manually based on location of adjacent genes and the presence of start and stop codons. Transfer RNA genes were located and folded by their proposed cloverleaf to confirm their secondary structures using ARWEN v.1.2^58^ and tRNAscan-SE v.1.21^59^, following the generalized invertebrate mitochondrial tRNA settings. Finally, our annotations were double-checked using MITOS WebServer^60^ under the invertebrate mitochondrial code.

### Concatenated alignment, divergences and positive Darwinian selection

With the exception of the *Atp8* gene that is not described for all the mitogenomes (Supplementary Table S1), the nucleotide sequences of the PCGs of the Pteriomorphia species (Table 1) were translated into amino acid sequences using the invertebrate mitochondrial genetic code, and aligned separately using the MAFFT platform of the TranslatorX multiple sequence alignment program^61^. Alignments were done using the L-INS-i option (accurate for alignment of ≤200 sequences) and default settings. The alignments were back-translated into the corresponding nucleotide sequences. This alignment procedure helped avoid the destruction of codons and displacement of nucleotides and aimed to obtain a reliably homologous region^61^. Ambiguously-aligned sites were removed using Gblocks v.0.19b implemented in TranslatorX^61^ with default settings. Nucleotide sequences for individual PCG alignments were concatenated before the phylogenetic analysis. These alignments were used to explore inter-specific divergences for each PCG within Mytilidae. Pairwise genetic distances were calculated using the Kimura’s two parameter and 1000 bootstrap replications for variance estimation with the program MEGA v.5.1^62^. Putative species limits was explored using the species delimitation pipeline implemented in the software Geneious V.9.1.3^49^.

To evaluate the nature of the selective forces acting on the mitogenomes we estimated the rate of nucleotide substitutions at non-synonymous (dN) relative to that at synonymous sites (dS), using the Datamonkey web server^63^. In HyPhy, implemented via the Datamonkey software package, the fixed effect likelihood (FEL), the single likelihood ancestor counting (SLAC), the fast, unconstrained Bayesian approximation (FUBAR), the mixed effects model of evolution (MEME), and the GA-Branch analyses were used^48^. These methods were applied to five datasets: i) all mtDNA-encoded PGCs; ii) the three subunits of the cytochrome c oxidase complex (Cox1-3); iii) the seven subunits of the NADH dehydrogenase complex (Nadh1-6; Nadh4l); iv) the cytochrome b subunit of the ubiquinol cytochrome c oxidoreductase complex (Cytb); and v) the *Atp6* and *Atp8* of the ATP synthase complex. In all analyses performed in Datamonkey, the most suited model of evolution for each data set, directly estimated on this web server, was used. Sites with p-values less than 0.05 for SLAC, FEL and MEME, posterior probability of more than 0.9 for FUBAR, were considered as being under selection. Sequences were screened for recombination to avoid misleading results in the selection analyses by mean of the Genetic Algorithms for Recombination Detection (GARD) in the Datamonkey web server^63^. No recombination was detected in the coding genes of any of the sequenced genomes.

### Phylogenetic reconstructions

Best Partition Scheme (BPS) analysis for the concatenated alignment was conducted with the program PartitionFinder^64^, using the Bayesian Information Criterion (BIC) and a heuristic search algorithm. This allowed us to compare different partition schemes for each codon position in each gene. A total of 39 data blocks were defined, following the criteria of one data block for each codon position in each gene. The BPS included seven subset partitions (Supplementary Table S4) with the models of molecular evolution used for both Bayesian (BI) and Maximum Likelihood (ML) analyses. ML inference was performed with RaxML v.7.2.6^65^, using the graphical interface RaxML-GUI^66^ invoking the GTRGAMMA and the rapid bootstrap option with 1000 replicates. In addition, a Bayesian inference (BI) MCMC analysis was conducted using MrBayes v.3.2^67^. The rate parameter was allowed to vary. Parameter estimation was “unlinked” for the shape of the gamma distribution used to model rate variation between sites, the substitution matrix, the proportion of invariable sites, and the estimation of state frequencies. Six Markov chains were used, and each chain was started from a random tree. The “temperature” parameter was set to a default value of 0.2. Two simultaneous runs of 10,000,000 generations were conducted, and trees were sampled every 1000 generations. To establish whether the Markov chains had reached a steady state, we plotted the –ln likelihood scores of sampled trees against generation time. Trees inferred prior to stationarity (i.e., lack of improvement in the likelihood score) were discarded as burn-in (first 10% of the sampled trees), and the remaining trees were used to construct a 50% majority-rule consensus tree.

### Data availability.

The data set supporting the results of this article is available in Dryad and can be found at http://dx.doi.org/10.5061/dryad.t6b62. All nucleotide sequences were deposited in the NCBI Genbankrepository. Accessions can be found in Table 1.

## Additional Information

**How to cite this article**: Gaitán-Espitia, J. D. *et al.* Mitogenomics of southern hemisphere blue mussels (Bivalvia: Pteriomorphia): Insights into the evolutionary characteristics of the *Mytilus edulis* complex. *Sci. Rep.* **6**, 26853; doi: 10.1038/srep26853 (2016).

## Supplementary Material

Supplementary Information

## Figures and Tables

**Table 1:** List of species used in this study.

Subclass	Order	Family	Species	GenBank
Pteriomorphia				
	Mytiloida			
		Mytilidae	*Mytilus chilensis*	KP100300
			*Mytilus platensis*	KP100301
			*Mytilus edulis*	AY484747
			*Mytilus galloprovincialis*	AY497292
			*Mytilus coruscus*	KJ577549
			*Mytilus trossulus*	AY823625
			*Mytilus californianus*	GQ527172
			*Brachidontes exustus*	KM233636
			*Musculista senhousia*	GU001954
			*Perna viridis*	JQ970425
	Arcoida			
		Arcidae	*Anadara sativa*	KF667521
			*Scapharca broughtonii*	AB729113
	Ostreoida			
		Ostreidae	*Crassostrea angulata*	FJ841965
			*Crassostrea ariakensis*	EU672835
			*Crassostrea gigas*	EU672831
			*Ostrea denselamellosa*	HM015199
			*Ostrea edulis*	JF274008
			*Ostrea lurida*	KC768038
			*Saccostrea mordax*	FJ841968
	Pectinoidea			
		Pectinidae	*Argopecten irradians*	DQ665851
			*Argopecten purpuratus*	KF601246
			*Chlamys farreri*	EU715252
			*Mimachlamys senatoria*	KF214684
			*Placopecten magellanicus*	DQ088274
	Pterioida			
		Pinnidae	*Atrina pectinata*	KC153059
			*Pinctada margaritifera*	HM467838
Protobranchia				
	Solemyoida			
		Solemyidae	*Solemya velum*	JQ728447
Palaeoheterodonta				
	Unionidae			
		Anodontinae	*Anodonta anatina*	KF030965
Heteroconchia				
	Veneroidea			
		Veneridae	*Meretrix meretrix*	GQ463598
	Lucinoidea			
		Lucinidae	*Loripes lacteus*	EF043341
		Lucinidae	*Lucinella divaricata*	EF043342
	Myoidea			
		Myidae	*Mya arenaria*	KJ755996

**Table 2:** Mitochondrial genome content and general features of the *Mytilus chilensis* and *M. platensis.*

	Direction	Lenght (bp)	Min	Max	Start codon	Stop codon	Anti-codon	AT%
*POR*	Forward	1173	1	1173				60.4
*tRNA-Tyr*	Forward	67	1174	1240			1205–1207	
*Cytb*	Forward	1308	1242	2549	ATG	TAG		60.6
*Cox2*	Forward	729	2552	3280	ATG	TAG		61.8
*tRNA-Lys*	Forward	69	3285	3353			3316–3318	
*tRNA-Met*	Forward	69	3357	3425			3390–3392	
*tRNA-Leu*	Forward	66	3429	3494			3458–3460	
*tRNA-Leu*	Forward	66	3498	3563			3527–3529	
*Nad1*	Forward	918	3726	4643	GTG	TAA		59.9
*tRNA-Val*	Forward	66	4644	4709			4675–4677	
*Nad4*	Forward	1308	4710	6017	ATG	TAA		59.4
*Cox3*	Forward	936	6021	6956	ATG	TAA		58.5
*tRNA-Ser*	Forward	63	6973	7035			7003–7005	
*tRNA-Met*	Forward	65	7038	7102			7070–7072	
*Nad2*	Forward	948	7106	8053	ATG	TAG		59.4
*tRNA-Arg*	Forward	65	8057	8121			8088–8090	
*tRNA-Trp*	Forward	68	8125	8192			8156–8158	
*tRNA-Ala*	Forward	64	8194	8257			8225–8227	
*tRNA-Ser*	Forward	66	8262	8327			8293–8295	
*tRNA-His*	Forward	64	8330	8393			8362–8365	
*tRNA-Pro*	Forward	65	8395	8459			8426–8428	
*Nad3*	Forward	351	8463	8813	ATG	TAA		58.7
*Atp8*	Forward	255	8832	9086	ATG	TAA		60.0
*Cox1*	Forward	1665	9089	10753	ATA	TAA		61.0
*Atp6*	Forward	717	10763	11479	ATG	TAG		61.1
*tRNA-Thr*	Forward	63	11484	11546			11515–11517	
*Nad4L*	Forward	282	11547	11828	ATG	TAA		64.6
*Nad5*	Forward	1707	11840	13546	ATA	TAA		62.3
*Nad6*	Forward	465	13546	14010	ATG	TAA		63.9
*tRNA-Phe*	Forward	68	14024	14091			14054–14056	
*s-rRNA*	Forward	946	14092	15037				64.0
*tRNA-Gly*	Forward	66	15038	15103			15071–15073	
*tRNA-Asn*	Forward	65	15104	15168			15135–15137	
*tRNA-Glu*	Forward	65	15169	15233			15199–15201	
*tRNA-Cys*	Forward	68	15235	15302			15268–15270	
*tRNA-Ile*	Forward	67	15303	15369			15334–15336	
*tRNA-Gln*	Forward	67	15385	15451			15414–15416	
*tRNA-Asp*	Forward	65	15457	15521			15487–15489	
*l-rRNA*	Forward	1244	15522	16765				64.5
