# Supplementary material for: Mitogenomics of southern hemisphere blue mussels (Bivalvia: Pteriomorphia): Insights into the evolutionary characteristics of the Mytilus edulis complex
Source: Sci Rep. 2016 May 31;6:26853. doi: 10.1038/srep26853 (PMC4886515; doi:10.1038/srep26853)
Supplement: Supplementary Information [file srep26853-s1.pdf]

Mitogenomics of southern hemisphere blue mussels (Bivalvia: Pteriomorphia): Insights into the evolutionary characteristics of the *Mytilus edulis* complex

Juan Diego Gaitán-Espitia[1,2*], Julian F. Quintero-Galvis[1,3], Andres Mesas[1,3] & Guillermo D'Elía[1]

[1] Instituto de Ciencias Ambientales y Evolutivas, Universidad Austral de Chile, Casilla 567 Valdivia, Chile.

[2] CSIRO Oceans and Atmosphere, GPO Box 1538, Hobart 7001, TAS, Australia

[3] Programa de Doctorado en Ciencias mención Ecología y Evolución, Facultad de Ciencias, Universidad Austral de Chile, Valdivia, Chile.

*** Corresponding author**: Juan Diego Gaitán-Espitia. Instituto de Ciencias Ambientales y Evolutivas, Facultad de Ciencias, Universidad Austral de Chile, Casilla 567 Valdivia, Chile. Tel: +56 63 221704; fax: +56 63 221673. Email address: juadiegaitan@gmail.com

Supplementary Figure Legends

Supplementary Figure S 1. Alignment of the Atp8 gene among species of the Family Mytilidae.

Supplementary Figure S 2. Mitochondrial genome syntenies of pteriomorph bivalves.

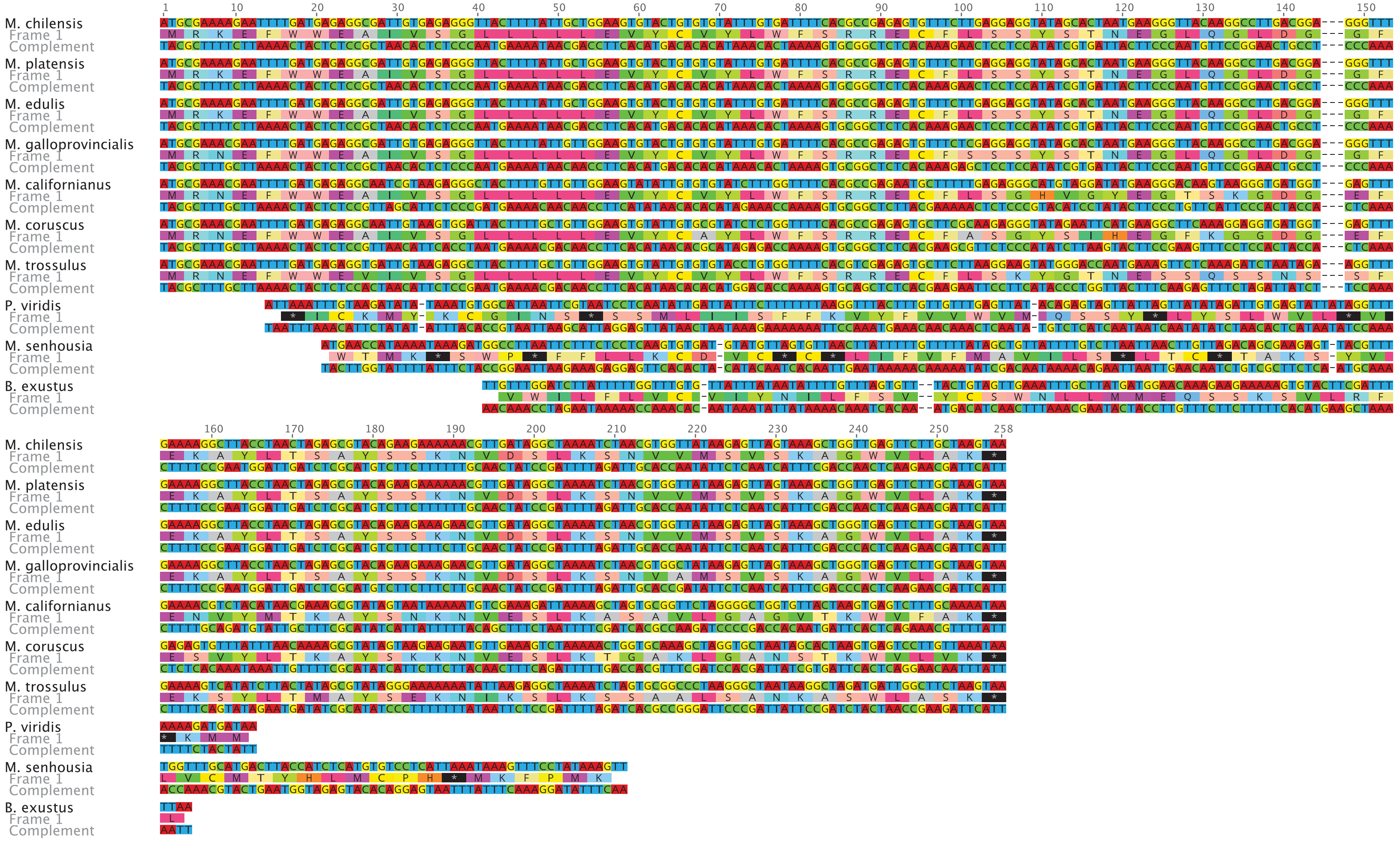
M. chilensis
M. platensis
M. edulis
M. galloprovincialis
M. californianus
M. coruscus
M. trossulus
P. viridis
M. senhousia
B. exustus
Frame 1
Complement

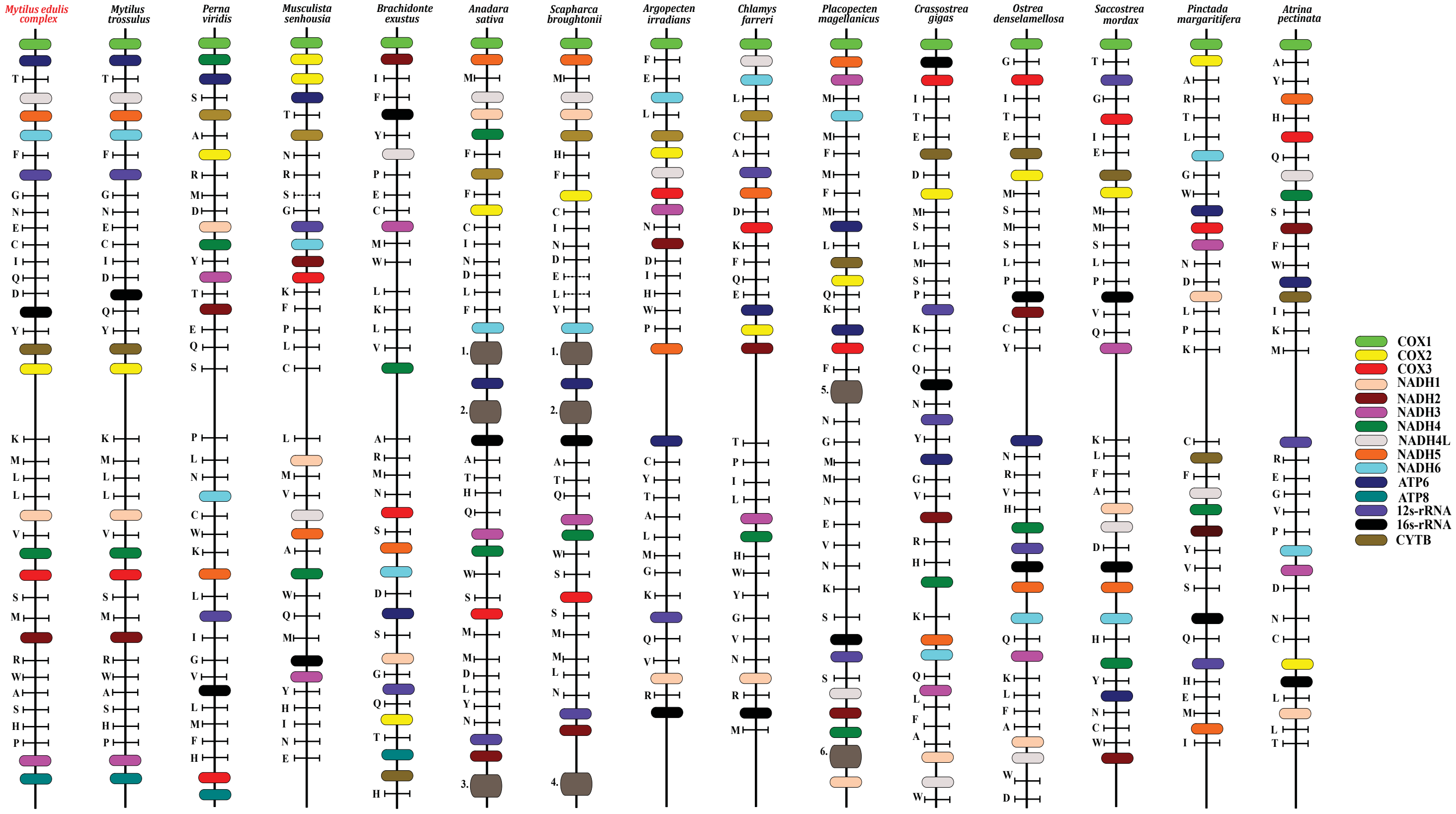

Mytilus edulis complex
Mytilus trossulus
Perna viridis
Musculista senhousia
Brachidonte exustus
Anadara sativa
Scapharca broughtonii
Argopecten irradians
Chlamys farreri
Placopecten magellanicus
Crassostrea gigas
Ostrea denselamellosa
Saccostrea mordax
Pinctada margaritifera
Atrina pectinata
COX1
COX2
COX3
NADH1
NADH2
NADH3
NADH4
NADH4L
NADH5
NADH6
ATP6
ATP8
12s-rRNA
16s-rRNA
CYTB

Supplementaty Table 1. Mitogenome size and general features in Bivalvia. Columns represent protein coding genes with their lengths (bp). Start and stop codons for the protein coding genes are enclosed in parentheses.

Subclase	Species	Total Size	ATP6	ATP8	COX1	COX2	COX3	CYTB	ND1	ND2	ND3	ND4	ND4L	ND5	ND6	%A	%C	%G	%T	%A-T	%G-C
Pteriomorphia	*Mytilus platensis*	16765	717 (ATG/AAA)	255 (ATG/AAG)	1665 (ATA/CAA)	729 (ATG/T)	937 (ATG/T)	1308 (ATG/ATA)	916 (GTG/GTG)	948 (ATG/ATT)	351 (ATG/GGT)	1308 (ATG/CCT)	282 (ATG/TCG)	1707 (ATA/ATA)	466 (ATG/AAA)	30.2	11.4	28.8	29.8	60	40
	Mytilus chilensis	16765	717 (ATG/T)	255 (ATG/AAG)	1665 (ATA/CAA)	729 (ATG/T)	937 (ATG/T)	1308 (ATG/ATA)	918 (GTG/GTG)	948 (ATG/ATT)	351 (ATG/GGT)	1308 (ATG/CTT)	282 (ATG/TCG)	1707 (ATA/ATA)	466 (ATG/AAA)	30.2	11.4	28.6	29.8	60	40
	Mytilus galloprovincialis	16744	717 (ATG/T)	255 (ATG/AAG)	1665 (ATA/CAA)	729 (ATG/T)	936 (ATG/GCT)	1308 (ATG/ATA)	916 (GTG/GTG)	948 (ATG/ATT)	351 (ATG/GGT)	1308 (ATG/CCT)	282 (ATG/TCG)	1705 (ATA/TTT)	465 (ATG/AAA)	29.4	12.2	29.4	29.0	58.4	41.6
	Mytilus trossulus	18652	717 (ATG/AGG)	255 (ATG/AAG)	1656 (ATG/CAA)	729 (ATG/AAC)	793 (ATG/GGA)	1308 (ATG/ATA)	918 (GTG/GTG)	948 (ATG/ATT)	351 (ATG/GGT)	1308 (ATG/CCC)	282 (ATG/TCG)	1707 (ATA/ATA)	465 (ATG/AAA)	31.8	12.2	25.9	30.2	62	38
	Mytilus californianus	16730	717 (ATG/T)	255 (ATG/AAA)	1656 (ATG/CAA)	729 (ATG/T)	936 (ATG/T)	1308 (ATG/ATA)	996 (ATA/GTG)	948 (ATG/TTG)	351 (ATG/GGC)	1308 (ATG/CCT)	282 (ATG/GTA)	1710 (ATA/ATA)	465 (ATG/AGG)	31.0	10.6	28.6	29.8	60.8	39.2
	Mytilus coruscus	16642	717 (ATG/AGT)	255 (ATG/AAA)	1656 (ATG/CAA)	729 (ATG/T)	936 (ATG/ACC)	1308 (ATG/ATA)	918 (ATA/GTA)	948 (ATG/CTA)	351 (ATG/GGA)	1305 (ATG/CCT)	282 (ATG/GTA)	1710 (ATT/ATG)	465 (ATG/AAA)	31.0	11.4	27.5	30.2	61.2	38.8
	Mytilus edulis	16740	717 (ATG/AGT)	255 (ATG/AAG)	1656 (ATG/CAA)	729 (ATG/T)	793 (ATG/GGA)	1194 (ATG/GTG)	924 (GTG/GTG)	945 (ATG/ATT)	351 (ATG/T)	1308 (ATG/CCT)	282 (ATG/TCG)	1707 (ATA/ATA)	477 (ATG/AGA)	29.8	11.4	29.4	29.4	59.2	40.8
	Musculista senhousia	20612	714 (ATG/T)	189 (ATG/GTT)	1584 (ATG/GTT)	813 (ATG/GGA)	855 (ATG/GAA)	1197 (ATA/T)	966 (ATG/TGA)	945 (ATA/ATG)	375 (ATG/TCT)	1329 (ATA/TGT)	216 (ATA/TTT)	1765 (ATA/TTT)	438 (ATA/AGC)	45.7	8.7	19.6	26.1	71.7	28.3
	Perna viridis	16014	714 (ATG/AAA)	150 (ATT/TGA)	1536 (ATG/T)	690 (GTG/T)	825 (ATG/TCA)	1152 (ATG/AGA)	933 (ATG/TTC)	1011 (ATA/TTT)	354 (ATG/ATT)	1339 (ATG/CCT)	274 (ATT/TAT)	1728 (ATG/ATT)	477 (ATG/ATC)	29.2	9.3	21.0	40.5	69.7	30.3
	Brachidontes exustus	16600	747 (ATG/T)	114 (TTG/TTT)	1605 (ATG/T)	708 (ATA/AGA)	855 (ATG/TCT)	1206 (GTG/GCT)	933 (ATG/AAT)	963 (ATT/GTA)	351 (ATG/GCT)	1299 (ATG/TTG)	273 (GTG/TCT)	1731 (ATG/TTA)	465 (ATG/GTT)	25.8	10.5	18.7	44.9	70.8	29.2
	Anadara sativa	48161	525 (ATA/T)		1584 (ATG/AGA)	666 (ATG/TTA)	759 (GTG/TTA)	1278 (GTG/CGT)	924 (ATA/T)	1008 (TTG/TTG)	360 (ATG/CTT)	948 (ATA/TTT)	285 (ATG/TGT)	1707 (ATA/TTC)	468 (ATA/GCT)	27.5	17.4	20.3	34.8	62.3	37.7
	Scapharca broughtonii	46985	525 (ATA/AAG)		1584 (ATG/AGA)	759 (ATG/TTA)	759 (GTG/TTA)	1278 (GTG/CGT)	948 (ATA/AAT)	567 (ATG/TTT)	360 (ATA/CTT)	1296 (ATA/TGT)	285 (ATG/TGT)	1707 (ATA/TTC)	468 (ATA/CGT)	25.3	8.8	22.5	43.4	68.8	31.2
	Argopecten irradians	16211	750 (GTG/T)		1602 (ATA/AGG)	699 (GTG/TCT)	825 (GTG/TTT)	1158 (TTG/TCT)	945 (GTG/GTT)	936 (TTG/CTG)	348 (ATA/GAT)	1317 (ATG/CTT)	275 (ATA/GGT)	1683 (ATG/TCT)	540 (ATG/GAT)	18.9	14.1	29.6	37.4	56.3	43.7
	Argopecten purpuratus	16266	636 (ATA/T)		1602 (ATG/AGG)	576 (ATG/TCT)	828 (ATA/TTT)	1158 (TTG/TCT)	828 (ATG/GTT)	993 (ATA/TTA)	348 (ATA/GAT)	1278 (ATG/CTT)	333 (ATG/GGA)	1683 (ATA/TCT)	528 (ATG/GAA)	17.6	14.8	28.6	39.0	56.6	43.4
	Chlamys farreri	21695	789 (ATG/GGG)		1644 (ATG/AGG)	671 (GTG/GTT)	852 (ATG/GTG)	1153 (ATG/AAG)	948 (ATG/CTT)	957 (ATA/TTT)	353 (ATA/TGC)	1284 (ATG/TTA)	318 (ATA/T)	1779 (ATG/GTT)	495 (ATA/AGA)	20.4	12.5	27.5	39.6	60	40
	Mimachlamys senatoria	17383	882 (ATG/AGG)	153 (ATG/TTG)	1626 (ATA/GTT)	714 (ATG/GAG)	864 (GTG/TAC)	1264 (ATG/TTA)	948 (TTG/TTA)	966 (ATG/ATT)	333 (ATG/GAT)	1245 (ATG/GTA)	324 (ATT/TGT)	1746 (ATG/AGT)	495 (ATG/AGG)	18.2	14.7	27.7	39.4	57.6	42.4
	Placopecten magellanicus	32115	750 (ATA/AAG)		1513 (ATG/GTG)	685 (ATG/TGG)	828 (ATA/GGT)	1188 (TTG/TAC)	942 (ATG/TTG)	966 (GTG/CTT)	372 (ATG/GAT)	1284 (ATG/ATC)	297 (GTG/GGG)	1860 (ATG/CCA)	567 (ATA/AGT)	14.8	13.4	34.9	48.3	51.7	48.3
	Crassostrea angulata	18408	684 (ATA/T)		1617 (ATG/TGT)	702 (ATG/GGA)	876 (ATG/TCT)	1239 (CTA/T)	936 (ATG/ATT)	999 (ATG/TAC)	351 (ATG/GAT)	1350 (ATG/ATG)	283 (ATG/GCT)	1671 (ATG/GTT)	474 (ATT/TTC)	27.6	14.7	22.1	35.6	63.2	36.8
	Crassostrea ariakensis	18414	675 (ATG/T)		1617 (ATA/TGT)	702 (ATG/GGG)	864 (ATG/TCT)	1203 (TTA/TTT)	936 (ATG/TTC)	999 (ATG/TAC)	351 (ATG/AAG)	1350 (ATG/GCA)	283 (ATA/GCT)	1671 (GTG/GTT)	480 (ATG/TAC)	26.8	15.2	22.5	35.5	62.3	37.7
	Crassostrea gigas	18225	684 (ATG/T)		1617 (ATG/TGT)	702 (ATG/GGA)	876 (ATG/TCT)	1239 (CTA/AAT)	936 (ATG/ATT)	999 (ATG/TAC)	351 (ATG/GAT)	1353 (ATG/ATG)	283 (ATG/GCT)	1671 (ATG/GTT)	477 (ATG/TTC)	24.6	15.1	22.0	38.2	62.9	37.1
	Ostrea denselamellosa	16277	675 (GTG/GCC)		1596 (ATG/ATC)	696 (ATG/GGA)	880 (ATG/TCG)	1161 (ATA/GAG)	933 (ATG/ATT)	999 (ATG/TAC)	354 (ATG/AGC)	1350 (ATG/TTT)	282 (GTG/GAA)	1671 (ATA/ATG)	468 (ATG/AAA)	22.9	16.2	23.8	37.1	60	40
	Ostrea edulis	16320	669 (ATA/GTA)		1566 (ATG/ATC)	693 (ATG/T)	888 (ATA/TCT)	1161 (ATC/GAG)	916 (ATG/ATA)	1008 (ATT/TAC)	354 (ATG/AGC)	1350 (ATG/TTT)	282 (ATG/GAG)	1548 (ATG/ATT)	468 (ATA/TCA)	26.5	13.0	20.9	39.5	66	34
	Ostrea lurida	16344	570 (ATG/T)	102 (ATA/TTA)	1611 (ATA/T)	693 (ATG/AAT)	880 (ATG/TCT)	1053 (ATG/GAG)	858 (ATA/ATG)	1005 (GTG/TTA)	353 (GTG/AGC)	1350 (ATG/ATT)	264 (ATA/GAG)	1671 (ATG/GTT)	474 (ATG/TCA)	26.5	13.0	20.9	39.5	66	34
	Saccostrea mordax	16532	678 (ATG/AAA)		1656 (ATG/CGC)	696 (ATG/T)	819 (ATG/TCT)	1173 (ATT/GTA)	948 (GTG/TTT)	975 (ATA/TAT)	354 (ATG/AAT)	1350 (ATG/CTT)	282 (GTG/GAA)	1671 (ATG/TTA)	468 (GTG/CCC)	20.9	13.9	25.2	40.0	60.9	39.1
	Atrina pectinata	16811	702 (GTG/CAG)		1560 (ATG/GCC)	687 (GTG/GAG)	816 (TTG/GTG)	1146 (ATT/CGT)	931 (ATG/ATT)	999 (ATG/TTT)	345 (TTG/TCT)	1359 (ATG/TTT)	303 (ATC/GTT)	1755 (ATG/TGT)	471 (ATG/ATT)	20.6	8.7	28.2	42.5	63.1	36.9
	Pinctada margaritifera	15680	642 (ATG/AAA)		1563 (ATG/GGG)	781 (ATG/GGG)	798 (ATG/TCC)	1146 (ATG/GAG)	936 (ATG/ATC)	970 (ATG/CTT)	408 (ATG/ATG)	1308 (ATG/T)	279 (ATG/GGG)	1611 (ATG/ACC)	432 (ATG/ACT)	21.1	10.2	29.2	39.6	60.6	39.4
Heteroconchia	*Meretrix meretrix*	19826	849 (ATG/AAA)		1644 (TTG/AAA)	1173 (ATG/AGT)	906 (ATG/TCT)	1218 (ATA/AAA)	903 (ATG/TTA)	1056 (ATG/GTG)	435 (ATG/TAC)	1362 (ATG/GTT)	300 (ATG/TTT)	1701 (ATA/TGA)	531 (ATA/CAG)	22.1	10.3	22.7	44.9	67	33
	Loripes lacteus	17321	645 (ATG/AAG)	118 (ATT/GAC)	1557 (ATA/AAA)	879 (ATA/GCT)	798 (ATG/TAT)	1140 (ATG/ATG)	867 (ATG/AAA)	1002 (ATG/GCT)	348 (GTG/GAG)	1350 (ATG/TTA)	327 (GTG/CAC)	1773 (ATA/GTG)	486 (ATG/TTG)	21.0	13.2	26.3	39.5	60.5	39.5
	Lucinella divaricata	18940	690 (ATA/GTA)	114 (ATT/TTT)	1554 (ATA/AAG)	930 (ATA/TCT)	798 (ATG/T)	1142 (ATG/GTT)	939 (ATG/AAA)	1038 (ATA/ATC)	342 (ATG/GAA)	1350 (ATG/TTA)	282 (GTG/CTG)	1671 (ATA/TTG)	483 (GTG/TTT)	21.3	13.0	25.4	40.3	61.6	38.4
	Mya arenaria	17947	729 (ATG/T)		1755 (ATA/TTA)	1434 (ATG/AAG)	861 (ATG/TTT)	1041 (ATA/TTA)	912 (ATG/TTT)	1065 (ATG/ACT)	423 (ATG/AAA)	1365 (ATA/CTT)	384 (ATA/TTT)	1518 (ATA/TTA)	546 (ATA/AAC)	25.8	12.0	23.6	38.7	64.5	35.5
Palaeoheterodonta	*Anodonta anatina*	15637	702 (ATA/T)	192 (ATG/AAG)	1542 (TTG/AGG)	681 (ATG/T)	780 (ATG/TCT)	1155 (ATT/TTC)	855 (TTG/TTA)	966 (ATG/TTC)	357 (ATG/GTG)	1347 (ATT/CTA)	279 (GTG/TTT)	1737 (GTG/CTT)	489 (ATT/TTC)	36.7	18.4	18.4	35.7	72.4	27.6
Protobranchia	*Solemya velum*	15660	696 (ATG/CAT)	165 (ATG/AAA)	1536 (ATG/GCC)	687 (ATG/GCG)	780 (ATG/AGG)	1123 (ATT/TGT)	924 (ATG/ATT)	1089 (ATG/TTA)	353 (ATG/AAA)	1344 (ATA/ATT)	297 (ATG/TGT)	1727 (ATG/AAA)	501 (ATA/GGT)	47.5	20.8	8.6	23.2	70.7	29.3

Supplementary Table 2. Codons that may be under positive selection

Codon	Gene	SLAC		FEL		MEME	FUBAR	
		dN-dS	p-value	dN-dS	p-value	p-value	dN-dS	Post. Pr.
246	*Cytb*	0.43	0.30	0.26	0.12	0.03	-0.08	0.47
275	*Cytb*	0.36	0.54	0.18	0.29	0.00	-0.51	0.14
383	*Cytb*	0.50	0.43	0.84	0.27	0.02	-0.21	0.47
384	*Cytb*	-0.12	0.72	5.70	0.04	0.00	2.20	0.56
1016	*Nad4*	0.53	0.28	0.36	0.09	0.02	-0.24	0.39
1059	*Nad4*	0.28	0.47	0.83	0.45	0.02	-0.33	0.32
1267	*Nad4*	0.36	0.54	0.17	0.35	0.01	-0.55	0.11
1362	*Nad4*	0.58	0.20	0.28	0.09	0.02	-0.05	0.52
1941	*Nad2*	0.76	0.20	1.18	0.04	0.10	0.21	0.72
2092	*Atp8*	0.28	0.51	0.23	0.48	0.03	-0.59	0.09
3029	*Nad5*	0.71	0.17	0.36	0.04	0.05	0.06	0.69
3753	*Nad6*	0.54	0.33	0.67	0.57	0.00	-0.41	0.34
3764	*Nad6*	0.42	0.43	0.23	0.31	0.00	-0.59	0.13
3766	*Nad6*	0.17	0.53	0.07	0.87	0.00	-0.35	0.18

Supplementary Table 3. dN/dS comparisons

Gene	Number of codons	dN	dS	Normalized dN/dS
Cytb	446	0.33	1.15	0.144
Cox2	290	0.41	1.16	0.179
Nad1	333	0.40	1.20	0.168
Nad4	450	0.67	1.61	0.222
Cox3	312	0.49	1.56	0.159
Nad2	342	0.67	1.57	0.227
Nad3	125	0.40	1.20	0.176
Atp8	95	0.76	1.38	0.212
Cox1	556	0.48	2.15	0.096
Atp6	254	0.55	1.28	0.193
Nad4L	94	0.84	1.72	0.232
Nad5	603	0.56	1.29	0.214
Nad6	182	0.81	1.74	0.262

Supplementary Table 4. Best Partition Scheme (BPS) and best-fit models of molecular evolution for the subsets partitions of the mitochondrial protein-coding genes alignment.

Subset	Model	Partition for codon position
1	GTR+I+G	*Cox1_1, Cox2_1, Cox3_1, Cytb_1, Nad1_1, Nad3_1*
2	GTR+G	*Cox1_2*
3	HKY+I+G	*Cox2_2, Cox3_2, Cytb_2, Nad1_2, Nad3_2*
4	GTR+I	*Cox1_3, Cox2_3, Cox3_3, Cytb_3, Nad1_3, Nad3_3*
5	GTR+I+G	*Nad2_1*, *Nad4_1*, *Nad4L_1*, *Nad5_1*, *Nad6_1*, *Atp6_1*
6	HKY+I+G	*Nad2_2*, *Nad4_2*, *Nad4L_2*, *Nad5_2*, *Nad6_2*, *Atp6_2*
7	GTR+I	*Nad2_3*, *Nad4_3*, *Nad4L_3*, *Nad5_3*, *Nad6_3*, *Atp6_3*

Supplementary Table 5. Estimates of Evolutionary Divergence between Sequences. The number of base substitutions per site from between sequences are shown. Standard error estimate(s) are shown in every other column. Analyses were conducted using the Kimura 2-parameter model. Codon positions included were 1st+2nd+3rd+Noncoding. All positions containing gaps and missing data were eliminated. There were a total of 10558 positions in the final dataset.

Brachidontes_exustus																		
Musculista_senhousia	0.650	0.011																
Mytilus_californianus	0.654	0.012	0.627	0.010														
Mytilus_chilensis	0.659	0.013	0.633	0.012	0.265	0.006												
Mytilus_coruscus	0.663	0.012	0.644	0.011	0.198	0.005	0.282	0.006										
Mytilus_edulis	0.663	0.012	0.637	0.012	0.271	0.006	0.021	0.001	0.289	0.007								
Mytilus_galloprovincialis	0.655	0.013	0.635	0.012	0.262	0.006	0.020	0.001	0.280	0.007	0.023	0.001						
Mytilus_platensis	0.660	0.013	0.634	0.012	0.264	0.006	0.002	0.000	0.282	0.006	0.021	0.001	0.021	0.001				
Mytilus_trossulus	0.678	0.013	0.637	0.011	0.266	0.007	0.189	0.004	0.287	0.007	0.197	0.004	0.186	0.004	0.189	0.004		
Perna_viridis	0.624	0.011	0.558	0.009	0.598	0.009	0.614	0.009	0.605	0.011	0.620	0.009	0.616	0.009	0.614	0.009	0.614	0.01
